# A side-sensitive synthetic chart for the multivariate coefficient of variation

**DOI:** 10.1371/journal.pone.0270151

**Published:** 2022-07-05

**Authors:** Wai Chung Yeong, Sok Li Lim, Zhi Lin Chong, Michael B. C. Khoo, Sajal Saha

**Affiliations:** 1 School of Mathematical Sciences, Sunway University, Petaling Jaya, Malaysia; 2 School of Accounting and Finance, Taylor University, Subang Jaya, Malaysia; 3 Department of Mathematical and Actuarial Sciences, Lee Kong Chian Faculty of Engineering and Science, Universiti Tunku Abdul Rahman, Kajang, Malaysia; 4 School of Mathematical Sciences, Universiti Sains Malaysia, Penang, Malaysia; 5 Department of Mathematics, International University of Business Agriculture and Technology, Dhaka, Bangladesh; Dartmouth College Geisel School of Medicine, UNITED STATES

## Abstract

Control charts for the coefficient of variations (*γ*) are receiving increasing attention as it is able to monitor the stability in the ratio of the standard deviation (*σ*) over the mean (*μ*), unlike conventional charts that monitor the *μ* and/or *σ* separately. A side-sensitive synthetic (SS) chart for monitoring *γ* was recently developed for univariate processes. The chart outperforms the non-side-sensitive synthetic (NSS) *γ* chart. However, the SS chart monitoring *γ* for multivariate processes cannot be found. Thus, a SS chart for multivariate processes is proposed in this paper. A SS chart for multivariate processes is important as multiple quality characteristic that are correlated with each other are frequently encountered in practical scenarios. Based on numerical examples, the side-sensitivity feature that is included in the multivariate synthetic *γ* chart significantly improves the sensitivity of the chart based on the run length performance, particularly in detecting small shifts (*τ*), and for small sample size (*n*), as well as a large number of variables (*p*) and in-control *γ* (*γ*_0_). The multivariate SS chart also significantly outperforms the Shewhart *γ* chart, and marginally outperforms the Multivariate Exponentially Weighted Moving Average (MEWMA) *γ* chart when shift sizes are moderate and large. To show its implementation, the proposed multivariate SS chart is adopted to monitor investment risks.

## 1. Introduction

Synthetic charts are among the charts proposed to increase the sensitivity for the detection of changes in process parameters. The first synthetic chart was proposed by Wu and Spedding [1] to monitor the process mean. Synthetic chart is different from the traditional Shewhart chart, where, unlike the traditional Shewhart chart which immediately classifies a process as out-of-control when a sample is not within the control limits, the synthetic chart only classifies a process as out-of-control when there are less than *L* samples between two successive samples that plot beyond the control limits. Note that the *L* samples must fall within the control limits. Synthetic charts were shown to outperform the traditional Shewhart chart. A recent review of synthetic-type charts is available in Rakitzis et al. [2]. Some of the more recent studies on synthetic charts are Lee et al. [3], Haq and Khoo [4], Hu et al. [5], Haq [6] and many others.

Synthetic charts are then proposed by Calzada and Scariano [7] to monitor *γ*, where shifts in the ratio of the standard deviation (*σ*) to the mean (*μ*) are monitored. This enables processes with an inconsistent *μ* and/or *σ* but a consistent ratio $\frac{\sigma }{\mu }$ to be monitored, and allows the detection of special cause(s) that shifts the ratio $\frac{\sigma }{\mu }$. The chart outperforms the Shewhart *γ* chart by Kang et al. [8], but not the EWMA *γ* chart by Castagliola et al. [9]. Numerous studies are extended for synthetic *γ* charts. Recently, Tran et al. [10] developed one with measurement errors, and Yeong et al. [11] designed synthetic *γ* charts to reduce cost.

For the synthetic charts discussed in the preceding paragraph, samples falling beyond the control limits can fall on either side of the control limits. This has led Yeong et al. [12] to develop a side-sensitive synthetic *γ* chart, where successive samples need to be on the same side of the limits. In this paper, this chart is referred to as the SS chart, while the non-side-sensitive synthetic *γ* chart is referred to as the NSS chart. The SS chart is shown to significantly outperform the NSS chart [12]. Yeong et al. [12] evaluated the SS chart through the average run length (*ARL*) and expected *ARL* (*EARL*) criteria. Subsequently, Yeong et al. [13] evaluated the SS chart through the median run length (*MRL*) and expected *MRL* (*EMRL*) criteria. Run lengths are commonly used to evaluate the performance of control charts, where run lengths measure the number of samples until the chart gives an out-of-control signal. There are two types of run lengths, the in-control run length which measures the number of samples collected until the chart gives a false out-of-control signal (i.e. the chart gives an out-of-control signal when the process is in-control) and the out-of-control run length which measures the number of samples until an out-of-control condition is detected by the chart. A chart is said to show good performance if it has a large in-control run length and a small out-of-control run length. Common measures of run length include the *ARL*, *MRL* and *SDRL*, where these measures evaluate the average, median and standard deviation of the run lengths, respectively. However, the *ARL*, *MRL* and *SDRL* requires the exact value of the shift size to be unknown, which is not possible in certain scenarios [9]. In these cases, the performance of the chart will be measured through the *EARL* and *EMRL*, which measures the expected value of the *ARL* and *MRL* over a range of shift sizes.

A SS chart for multivariate processes is not available. To fill this gap, a multivariate SS chart is proposed in this paper. Multivariate charts are more useful in practice as most processes usually involve several quality characteristics which are correlated to each other, hence, they have to be jointly monitored. Dubious conclusions will be obtained if different univariate charts are used to monitor these quality characteristics, as the correlation between these quality characteristics are ignored. The first multivariate *γ* chart can be found in Yeong et al. [14]. Subsequently, Lim et al. [15] proposed the multivariate run sum *γ* chart; Abbasi and Adegoke [16] studied the phase-I implementation of multivariate *γ* charts; Khaw et al. [17], Chew et al. [18], Nguyen et al. [19] and Ayyoub et al. [20] varied the charting parameters of multivariate *γ* charts; Khatun et al. [21] proposed multivariate *γ* charts for short production runs; Giner-Bosch et al. [22], and Haq and Khoo [23] developed a multivariate EWMA (MEWMA) chart to monitor *γ*; Chew et al. [24] and Chew et al. [25] proposed multivariate run rules *γ* charts; finally, Ayyoub et al. [26], Ayyoub et al. [27] and Nguyen et al. [28] proposed multivariate *γ* charts that consider measurement errors.

Although several multivariate charts are available in the literature to monitor *γ*, a multivariate SS chart cannot be found. A multivariate SS chart will be proposed in this paper. Section 2 gives a list of notations and abbreviations that are used throughout the paper. Next, Section 3 gives a description of the properties of the sample *γ* $\left(\hat{\gamma }\right)$. Subsequently, a description of how the proposed multivariate SS chart operates is provided in Section 4, together with the formulae for the *ARL*, standard deviation of the run length (*SDRL*) and *EARL*, and the algorithms to optimize its performance. These algorithms are implemented on several numerical examples in Section 5, while Section 6 compares the multivariate SS chart with the multivariate NSS, MEWMA and Shewhart *γ* charts. Next, Section 7 shows the implementation of the proposed chart through an illustrative example, followed by the conclusion in Section 8.

### 2. List of abbreviations and notations

Table 1 shows the list of abbreviations and notations that are used throughout the paper.

**Table 1:** List of abbreviations and notations.

Abbreviations / Notations	Description
*μ*	Mean
**μ**	Mean vector
$\bar{X}$	Sample mean vector
*σ*	Standard deviation
**Σ**	Covariance matrix
**S**	Sample covariance matrix
*γ*	Coefficient of variation
$\hat{\gamma }$	Sample coefficient of variation
*γ*_0_	In-control coefficient of variation
*γ*_1_	Out-of-control coefficient of variation
*τ*	Shift size
$\mu ({\hat{\gamma }}^{2})$	Mean of ${\hat{\gamma }}^{2}$
$\mu_{0}({\hat{\gamma }}^{2})$	In-control mean of ${\hat{\gamma }}^{2}$
$\sigma ({\hat{\gamma }}^{2})$	Standard deviation of ${\hat{\gamma }}^{2}$
$\sigma_{0}({\hat{\gamma }}^{2})$	In-control standard deviation of ${\hat{\gamma }}^{2}$
$\mu_{1}^{'}(F')$	First moment of *F*’
${\tilde{\mu }}_{1}^{'}(F')$	Adjusted first moment of *F*’ for *p* = 2
$\mu_{2}^{'}(F')$	Second moment of *F*’
${\tilde{\mu }}_{2}^{'}(F')$	Adjusted second moment of *F*’ for *p*∈{2,3,4}
*K*	Control limit coefficient
*L*	Threshold for the CRL sub-chart
*n*	Sample size
*p*	Number of variables
*ARL*	Average run length
*ARL*_0_	In-control average run length
*ARL*_1_	Out-of-control average run length
CRL	Conforming run length
*EARL*	Expected average run length
*EMRL*	Expected median run length
EWMA	Exponentially weighted moving average
*LCL*	Lower control limit
MEWMA	Multivariate exponentially weighted moving average
*MRL*	Median run length
NSS	Non-side-sensitive synthetic chart
*SDRL*	Standard deviation of the run length
*SDRL*_0_	In-control standard deviation of the run length
*SDRL*_1_	Out-of-control standard deviation of the run length
SS	Side-sensitive synthetic chart
*UCL*	Upper control limit
0	Sample where $LCL<\hat{\gamma }<UCL$
1	Sample where $\hat{\gamma }<LCL$
1	Sample where $\hat{\gamma }>UCL$

### 3. Properties of the sample multivariate coefficient of variation $\left(\hat{\gamma }\right)$

Let **X** = (*X*_1_,*X*_2_,…,*X*_*p*_)^*T*^ be *p* quality characteristics from a multivariate normal distribution with mean vector **μ**^**T**^ = (*μ*_1_,*μ*_2_,…,*μ*_*p*_) and covariance matrix $\Sigma =(\begin{array}{cccc} \sigma_{11} & \sigma_{12} & \ldots & \sigma_{1p}\\ \sigma_{12} & \sigma_{22} & \ddots & \sigma_{2p}\\ \vdots & \ddots & \ddots & \vdots \\ \sigma_{1p} & \sigma_{2p} & \cdots & \sigma_{pp} \end{array})$. The multivariate *γ* is defined as [29]

$$\gamma ={\left(\mu^{T}\Sigma^{-1}\mu \right)}^{-\frac{1}{2}}.$$ (1)

To monitor *γ*, samples of size *n* are collected and measured at regular intervals. We denote the measurement of the *j*^th^ quality characteristic of the *i*^th^ unit in the sample as *X*_*ij*_. The sample mean vector can be obtained as

$$\bar{X}={\left(\frac{1}{n}\sum_{i=1}^{n}X_{i1},\frac{1}{n}\sum_{i=1}^{n}X_{i2},\ldots ,\frac{1}{n}\sum_{i=1}^{n}X_{ip}\right)}^{T},$$ (2)

while the sample covariance matrix is given as

$$S=\frac{1}{n-1}\sum_{i=1}^{n}(X_{i}-\bar{X}){\left(X_{i}-\bar{X}\right)}^{T},$$ (3)

where **X**_*i*_ = (*X*_*i*1_,…,*X*_*ip*_)^*T*^. The sample *γ* $\left(\hat{\gamma }\right)$ can then be computed as

$$\hat{\gamma }={\left({\bar{X}}^{T}S^{-1}\bar{X}\right)}^{-\frac{1}{2}},$$ (4)

where $\hat{\gamma }$ is the (biased) natural estimator of *γ*. From Yeong et al. [14],

$$\frac{n(n-p)}{(n-1)p{\hat{\gamma }}^{2}}∼F(p,n-p,\frac{n}{\gamma^{2}}),$$ (5)

i.e. $\frac{n(n-p)}{(n-1)p{\hat{\gamma }}^{2}}$ follows a non-central *F* distribution with *p* and (*n*−*p*) degrees of freedom (df) and a non-centrality parameter (ncp) of $\frac{n}{\gamma^{2}}$, with *n*>*p*. From Eq (5), the cumulative distribution function (cdf) for $\hat{\gamma }$ is obtained as

$$F_{{\hat{\gamma }}^{2}}(x|n,p,\gamma )=1-F_{F}(\frac{n(n-p)}{(n-1)px}|p,n-p,\frac{n}{\gamma^{2}}),$$ (6)

where $F_{F}(.|p,n-p,\frac{n}{\gamma^{2}})$ is the cdf for the non-central *F* distribution in Eq (5).

By letting $F'=\frac{(n-1)p{\hat{\gamma }}^{2}}{n(n-p)}$, it follows that ${\hat{\gamma }}^{2}=\frac{n(n-p)}{(n-1)p}F'$. Thus, the mean and standard deviation of ${\hat{\gamma }}^{2}$ can be obtained through the first and second moments of *F*’ as follows

$$\mu ({\hat{\gamma }}^{2})=\frac{n(n-p)}{(n-1)p}\mu_{1}^{'}(F')$$ (7)

and

$$\sigma ({\hat{\gamma }}^{2})=\frac{n(n-p)}{(n-1)p}\sqrt{\mu_{2}^{'}(F')-{\left(\mu_{1}^{'}(F')\right)}^{2}},$$ (8)

where $\mu_{1}^{'}(F')$ and $\mu_{2}^{'}(F')$ are the first and second moments of *F*’. Since $F'={\left(\frac{n(n-p)}{(n-1)p{\hat{\gamma }}^{2}}\right)}^{-1},$ from Eq (5) *F*’ is a non-central *F* variable with *n*−*p* and *p* df and ncp of 0 and $\frac{n}{\gamma^{2}}$, i.e., $F'∼F(n-p,p,0,\frac{n}{\gamma^{2}})$. From Giner-Bosch et al. [22], the first and second moments of *F*’ can be obtained as follows:

$$\mu_{1}^{'}(F')=\frac{p}{2}C(\frac{p}{2}-1,-\frac{n}{2\gamma^{2}}),$$ (9)

$$\mu_{2}^{'}(F')=\frac{p^{2}}{4(p-4)}(\frac{2}{n-p}+1)(2-(\frac{n}{\gamma^{2}}+p-4)C(\frac{p}{2}-1,-\frac{n}{2\gamma^{2}})),$$ (10)

where *C*(*a*,*z*) in Eqs (9) and (10) is obtained as

$$C(a,z)=\frac{1}{a+\frac{-az}{a+1+\frac{z}{a+2+\frac{-(a+1)z}{a+3+\frac{2z}{a+4+\frac{-(a+2)z}{a+5+\frac{3z}{a+6+\ldots }}}}}}}.$$ (11)

where *C*(*a*,*z*) will converge with sufficient accuracy with 300 nested fractions [22]. Thus, this paper will adopt the same number of nested fractions.

Note that $\mu_{1}^{'}(F')$ is undefined for *p*≤2 and $\mu_{2}^{'}(F')$ is undefined for *p*≤4 [22]. For these cases, Giner-Bosch et al. [22] suggested the following alternative versions of $\mu_{1}^{'}(F')$ and $\mu_{2}^{'}(F')$:

$${\tilde{\mu }}_{1}^{'}(F')=\frac{1}{1-\varepsilon }\int_{0}^{u_{0}}uf_{F'}(u)du$$ (12)

and

$${\tilde{\mu }}_{2}^{'}(F')=\frac{1}{1-\varepsilon }\int_{0}^{u_{0}}u^{2}f_{F'}(u)du,$$ (13)

where *ε* is a small value (for example 10^−4^), $u_{0}=F_{F'}^{-1}(1-\varepsilon )$, $F_{F'}^{-1}(.)$ is the inverse cdf for *F*’, and *f*_*F*’_(.) is the probability density function (pdf) for *F*’. Eqs (12) and (13) can be numerically integrated [30].

For *p* = 2, $\mu ({\hat{\gamma }}^{2})$ and $\sigma ({\hat{\gamma }}^{2})$ can be computed from Eqs (7) and (8) by replacing $\mu_{1}^{'}(F')$ and $\mu_{2}^{'}(F')$ with ${\tilde{\mu }}_{1}^{'}(F')$ and ${\tilde{\mu }}_{2}^{'}(F')$, respectively, while for *p*∈{3,4}, since $\mu_{1}^{'}(F')$ is finite, only $\mu_{2}^{'}(F')$ needs to be replaced with ${\tilde{\mu }}_{2}^{'}(F')$ in Eq (8) to obtain $\sigma ({\hat{\gamma }}^{2})$.

### 4. A multivariate side-sensitive synthetic chart for monitoring ${\hat{\gamma }}^{2}$

This section describes the multivariate SS chart for ${\hat{\gamma }}^{2}$. The same approach as that in Yeong et al. [12] is adopted, but by adapting it for ${\hat{\gamma }}^{2}$ of multivariate processes, since the SS chart proposed by Yeong et al. [12] monitors $\hat{\gamma }$ for univariate processes.

The synthetic *γ* chart is made up of the Shewhart *γ* and conforming run length (CRL) sub-charts. For the Shewhart sub-chart of the NSS chart, when $\hat{\gamma }>UCL$ or $\hat{\gamma }<LCL$, where *UCL* and *LCL* are the upper and lower control limits, then that sample is non-conforming; conversely, it is conforming. The CRL sub-chart then defines the CRL as the number of conforming samples between two successive non-conforming samples, inclusive of the most recent non-conforming sample. For example, if there are five conforming samples between two successive non-conforming samples, then CRL = 6. If CRL≤*L*, with *L* being a threshold set by the user, the process is considered to have gone out-of-control. In other words, if there are less than *L* conforming samples between two successive non-conforming samples, the chart will produce an out-of-control signal. The SS chart includes an additional feature where successive non-conforming samples must belong to the same side of the centreline (*CL*). Hence, if the first non-conforming sample is above the *UCL* (below the *LCL*), then only samples that are above the *UCL* (below the *LCL*) are non-conforming.

**Fig 1:**
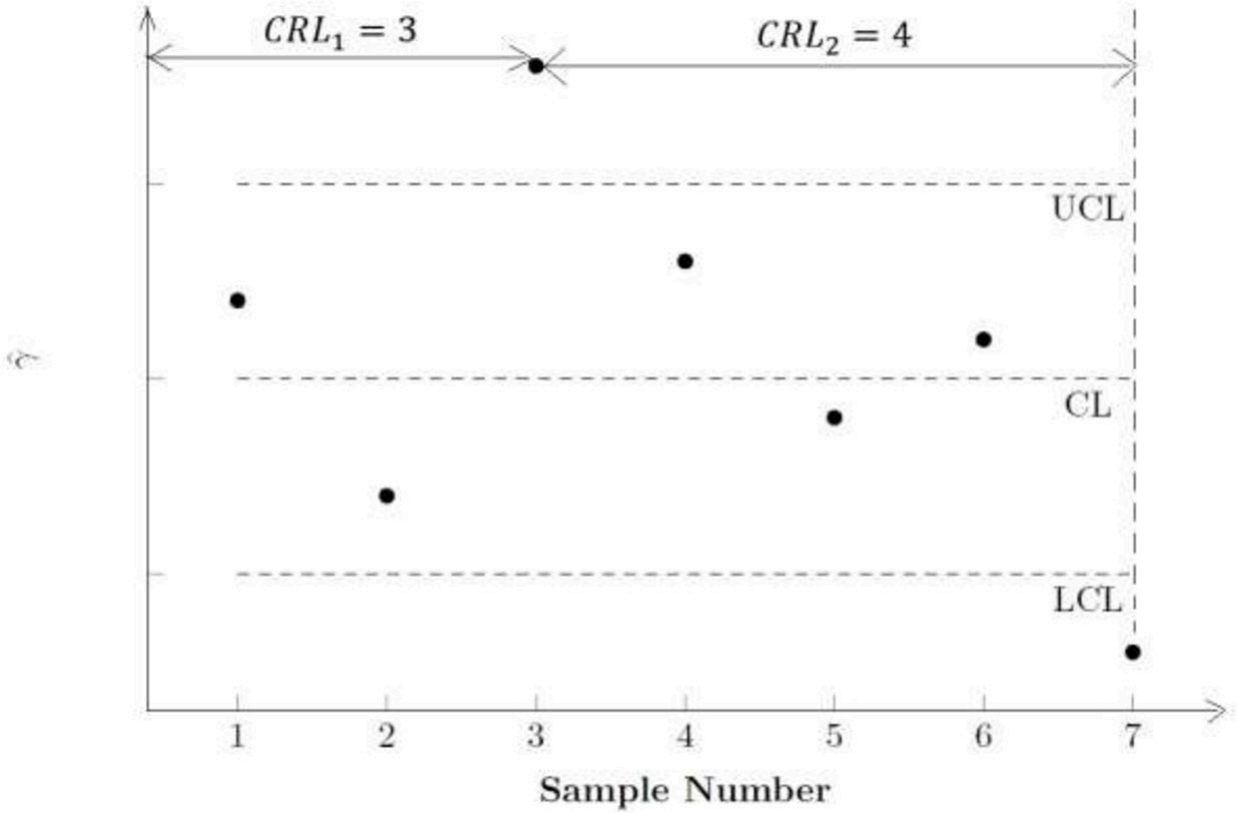
The *CRL* sub–chart of the Non–side–sensitive Synthetic–*γ* (NSS) chart.

**Fig 2:**
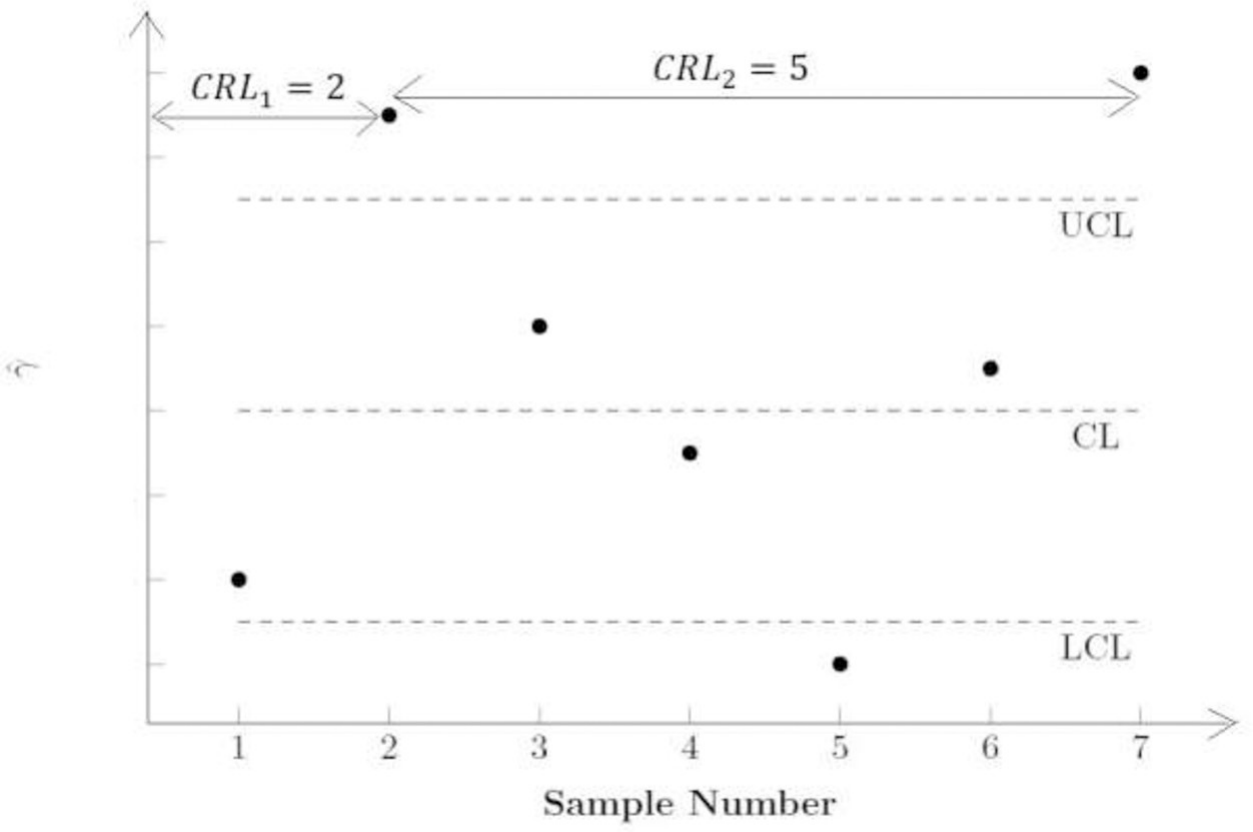
The *CRL* sub–chart of the Side–sensitive Synthetic–*γ* (SS) chart.

Figs 1 and 2 illustrate the difference between the NSS and SS charts. From Fig 1, Sample 3 is the first non-conforming sample, and it falls above the *UCL*, while Sample 7 is the second non-conforming sample, and it falls below the *LCL*. For the NSS chart, both samples are considered to be non-conforming samples, although they fall on different sides of the *CL*. Thus the CRL = 4. By comparison, for the SS chart in Fig 2, although Samples 2, 5 and 7 falls outside the region between *LCL* and *UCL*, the CRL = 5. This is because the first sample to fall outside the region between *LCL* and *UCL*, Sample 2, falls above the *UCL*. Although Sample 5 falls outside the region between *LCL* and *UCL*, it is not considered to be a non-conforming sample as it falls below the *LCL*, which is on the opposite side of the *CL* from Sample 2. Instead, the next non-conforming sample is Sample 7, since similar with Sample 2, it also falls above the *UCL*. As a result, CRL = 5. In short, successive non-conforming samples for the SS chart needs to fall on the same side of the *CL*, whereas successive non-conforming samples for the NSS chart do not have to fall on the same side of the *CL*.

The proposed multivariate SS chart monitors ${\hat{\gamma }}^{2}$, instead of $\hat{\gamma }$, due to the availability of the mean and standard deviation of ${\hat{\gamma }}^{2}$ from Giner-Bosch et al. [22]. The following are the *LCL* and *UCL* of the proposed multivariate SS chart

$$LCL=\mu_{0}({\hat{\gamma }}^{2})-K\sigma_{0}({\hat{\gamma }}^{2})$$ (14)

and

$$UCL=\mu_{0}({\hat{\gamma }}^{2})+K\sigma_{0}({\hat{\gamma }}^{2}),$$ (15)

where $\mu_{0}({\hat{\gamma }}^{2})$ and $\sigma_{0}({\hat{\gamma }}^{2})$ are the in-control mean and standard deviation of ${\hat{\gamma }}^{2}$ which is obtained from Eqs (7) and (8), respectively, by evaluating the first and second moments of *F*’ by letting $\gamma^{2}=\gamma_{{}_{0}}^{2}$, with *γ*_0_ being the in-control value of *γ*, while *K* is the control limit coefficient that controls the width of the region between *LCL* and *UCL*. The last two paragraphs of this section describe the methodology in determining the value of *K*.

A Markov chain approach similar to that by Yeong et al. [12] is adopted to obtain the *ARL*, *SDRL* and *EARL* values, but modified for the case of multivariate processes. The states of the Markov chain are defined as in Yeong et al. [12] based on a string of *L* successive samples, where each sample is defined as either 0, 1 or 1, which denote samples between the *LCL* and *UCL*, samples below the *LCL* and samples above the *UCL*, respectively. The states of the Markov chain are defined as follows:

State 1: 100..0

State 2: 010..0

State 3: 001..0

⋮

State *L*: 000..1

State *L* + 1: 00..00

State *L* + 2: 0..001

State *L* + 3: 0..010

⋮

State 2*L*: 010..0

State 2*L* + 1: 100..0

State 2*L* + 2: Signaling state (i.e. the state where the chart signals an out-of-control condition when *CRL*≤*L*)

A (2*L*+2)×(2*L*+2) transition probability matrix is then formed as follows:

$$\begin{array}{l} \begin{array}{cccccccccccc} \;\;1 & 2 & \cdots & L-1 & L & L+1 & L+2 & L+3 & L+4 & \cdots & 2L+1 & 2L+2 \end{array}\\ P=\left(\begin{array}{cc} Q & r\\ 0^{T} & 1 \end{array}\right)=\begin{array}{c} 1\\ 2\\ 3\\ \vdots \\ L\\ L+1\\ L+2\\ L+3\\ \vdots \\ 2L\\ 2L+1\\ 2L+2 \end{array}\left(\begin{array}{cccccccccccc} 0 & 0 & \cdots & 0 & 0 & A & B^{+} & 0 & 0 & \cdots & 0 & B^{-}\\ A & 0 & \cdots & 0 & 0 & 0 & B^{+} & 0 & 0 & \cdots & 0 & B^{-}\\ 0 & A & \cdots & 0 & 0 & 0 & B^{+} & 0 & 0 & \cdots & \cdots & B^{-}\\ \cdots & \cdots & \cdots & \cdots & \cdots & \cdots & \cdots & \cdots & \cdots & \cdots & \cdots & \cdots \\ 0 & 0 & \cdots & A & 0 & 0 & B^{+} & 0 & 0 & \cdots & 0 & B^{-}\\ 0 & 0 & \cdots & 0 & B^{-} & A & B^{+} & 0 & 0 & \cdots & 0 & 0\\ 0 & 0 & \cdots & 0 & B^{-} & 0 & 0 & A & 0 & \cdots & 0 & B^{+}\\ 0 & 0 & \cdots & 0 & B^{-} & 0 & 0 & 0 & A & \cdots & 0 & B^{+}\\ \cdots & \cdots & \cdots & \cdots & \cdots & \cdots & \cdots & \cdots & \cdots & \cdots & \cdots & \cdots \\ 0 & 0 & \cdots & 0 & B^{-} & 0 & 0 & 0 & 0 & \cdots & A & B^{+}\\ 0 & 0 & \cdots & 0 & B^{-} & A & 0 & 0 & 0 & \cdots & 0 & B^{+}\\ 0 & 0 & \cdots & 0 & 0 & 0 & 0 & 0 & 0 & \cdots & 0 & 1 \end{array}\right), \end{array}$$ (16)

where

$$\begin{array}{l} A=P(LCL<{\hat{\gamma }}^{2}<UCL)\\ \;=F_{F}(\frac{n(n-p)}{(n-1)(LCL)p}|p,n-p,\frac{n}{\gamma^{2}})-F_{F}(\frac{n(n-p)}{(n-1)(UCL)p}|p,n-p,\frac{n}{\gamma^{2}}) \end{array}$$ (17)

$$\begin{array}{l} B^{+}=P({\hat{\gamma }}^{2}>UCL)\\ \;=F_{F}(\frac{n(n-p)}{(n-1)(UCL)p}|p,n-p,\frac{n}{\gamma^{2}}) \end{array}$$ (18)

$$\begin{array}{l} B^{-}=P(\hat{\gamma }<LCL)\\ \;=1-F_{F}(\frac{n(n-p)}{(n-1)(LCL)p}|p,n-p,\frac{n}{\gamma^{2}}). \end{array}$$ (19)

The *ARL* and *SDRL* can be obtained from the Markov chain in Eq (16) by evaluating the expected and standard deviation for the number of transitions until the Markov chain reaches the out-of-control state (State 2*L*+2), as follows

$$ARL=q^{T}{\left(I-Q\right)}^{-1}1$$ (20)

and

$$SDRL=\sqrt{2q^{T}{\left(I-Q\right)}^{-2}Q1-ARL^{2}+ARL},$$ (21)

where **q** is a (2*L*+1)×1 vector of initial transient state probabilities, **I** is the identity matrix, and **1** is a vector of ones. The derivations for Eqs (20) and (21) are shown in Yeong et al. [12]. A zero-state condition is considered, so the (*L*+2)^th^ element of **q** is one, and all other elements are zeros, in order to give the proposed chart a head-start. The out-of-control *ARL* (*ARL*_1_) and *SDRL* (*SDRL*_1_) are obtained by substituting *γ* = *γ*_1_ = *τγ*_0_ into Eqs (20) and (21), where *τ*, *γ*_1_ and *γ*_0_ denote the shift size, out-of-control *γ* and in-control *γ*, respectively, while the in-control *ARL* (*ARL*_0_) and *SDRL* (*SDRL*_0_) are computed by substituting *γ* = *γ*_0_ into Eqs (20) and (21).

To evaluate the *ARL* and *SDRL*, the exact value of *τ* must be known. This is not possible in some practical scenarios [9]. For such cases, the *EARL* is adopted to measure the performance of the chart, as follows:

$$EARL=\int_{\tau_{\min}}^{\tau_{\max}}f_{\tau }(\tau )ARL(\tau ,\gamma_{0},n,p,L,K)d\tau ,$$ (22)

with *f*_*τ*_(*τ*) being the pdf of *τ*. In most scenarios, there is a lack of available data to estimate the actual distribution of *τ*, hence, this paper assumes that *τ* follows a uniform distribution over the interval (*τ*_min_,*τ*_max_) [9]. To evaluate the integral in Eq (22), the Gauss-Legendre quadrature is adopted [31].

Two approaches will be adopted so that the optimal charting parameters (L*,K*) are obtained. Firstly, (L*,K*) is obtained to minimize the *ARL*_1_ for pre-determined values of (*τ*,*n*,*p*,*γ*_0_), subject to satisfying constraints in the *ARL*_0_, i.e.,

$$(L*,K*)=\underset{\left(L,K\right)}{\arg\;\min}ARL_{1}(\tau ,\gamma_{0},n,p,L,K),$$ (23)

subject to

$$ARL_{0}(\gamma_{0},n,p,L,K)=\xi ,$$ (24)

where *ξ* is the pre-determined *ARL*_0_ value. In this paper, we consider *L*∈{1,2,…,100}, and for each of these values of *L*, the value of *K* that satisfies Eq (24) will be obtained through numerical methods. Among all the combinations of (*L*,*K*), the combination with the smallest *ARL*_1_ will be the optimal (L*,K*). The optimal (LCL*,UCL*) is then obtained from Eqs (14) and (15). Subsequently, the smallest *ARL*_1_ value is obtained by substituting (L*,LCL*,UCL*) into Eq (20).

In the second approach, (L*,K*) is obtained based on minimizing the *EARL* value for pre-determined values of (*τ*_min_,*τ*_max_,*n*,*p*,*γ*_0_), subject to satisfying constraints in the *ARL*_0_. A similar approach to that described in the preceding paragraph is adopted, with the exception that (L*,K*) minimizes the *EARL* value, and the shift is the range (*τ*_min_,*τ*_max_), instead of an exact value *τ*.

### 5. Numerical examples

The optimal SS chart for several numerical examples will be obtained in this section. As described in Section 3, two approaches will be adopted, where the first approach minimizes *ARL*_1_, and the second minimizes *EARL*. In both of these approaches, the *ARL*_0_ constraint is set as 370.4. In this paper, we consider *p*∈{2,3,5,8}, *τ*∈{1.10,1.20,1.25,1.50,2.00} and *γ*_0_∈{0.10,0.20,0.30,0.50}. For *p*∈{2,3}, *p* = 5 and *p* = 8, we consider *n*∈{5,10}, *n*∈{6,10} and *n*∈{10,15}, respectively. Different *n* are considered for different *p* as *n* needs to be larger than *p*. Tables 2 and 3 show the optimal charting parameters and their *ARL*_1_ and *SDRL*_1_ values for *p*∈{2,3} and *p*∈{5,8}, respectively.

**Table 2:** Optimal charting parameters and the corresponding ARL1 and SDRL1 values of the multivariate SS Chart for *p*∈{2,3}, *n*∈{5,10}, *τ*∈{0.10,1.20,1.25,1.50,2.00} and *γ*_0_∈{0.10,0.20,0.30,0.50}.

*τ*	*γ*_0_ = 0.10				*γ*_0_ = 0.20				*γ*_0_ = 0.30				*γ*_0_ = 0.50			
	L*	K*	*ARL*_1_	*SDRL*_1_	L*	K*	*ARL*_1_	*SDRL*_1_	L*	K*	*ARL*_1_	*SDRL*_1_	L*	K*	*ARL*_1_	*SDRL*_1_
	*p* = 2															
	*n* = 5															
1.10	47	3.60	74.72	97.85	48	3.70	76.78	100.55	49	3.85	80.43	105.35	49	3.95	94.08	123.16
1.20	27	3.37	26.36	33.71	28	3.46	27.47	35.17	29	3.58	29.48	37.85	31	3.62	37.39	48.49
1.25	22	3.29	17.90	22.41	22	3.35	18.73	23.56	23	3.46	20.25	25.59	25	3.48	26.33	33.80
1.50	10	2.95	5.36	5.92	10	2.99	5.66	6.36	11	3.09	6.23	7.05	13	3.04	8.55	10.14
2.00	5	2.64	2.10	1.75	5	2.67	2.22	1.92	6	2.78	2.45	2.16	7	2.65	3.38	3.39
*τ*	*n* = 10															
1.10	31	3.04	44.09	57.46	32	3.11	46.07	60.07	33	3.23	49.46	64.55	35	3.54	60.80	79.48
1.20	15	2.80	12.50	15.49	16	2.87	13.28	16.50	17	2.97	14.66	18.33	19	3.24	19.59	24.96
1.25	12	2.73	8.07	9.57	12	2.77	8.61	10.32	13	2.87	9.56	11.57	15	3.12	13.01	16.20
1.50	5	2.43	2.41	2.19	5	2.46	2.56	2.42	6	2.57	2.84	2.72	7	2.75	3.88	4.11
2.00	3	2.25	1.22	0.55	3	2.27	1.27	0.63	3	2.30	1.35	0.78	4	2.48	1.69	1.23
*τ*	*p* = 3															
	*n* = 5															
1.10	53	3.87	88.50	115.98	54	3.95	90.70	118.88	56	4.08	94.62	124.03	56	4.17	109.31	143.16
1.20	34	3.66	34.01	43.73	34	3.72	35.34	45.54	35	3.82	37.75	48.78	38	3.89	47.33	61.58
1.25	27	3.55	23.67	30.00	28	3.62	24.71	31.37	29	3.72	26.61	33.91	31	3.75	34.28	44.27
1.50	13	3.21	7.40	8.52	14	3.27	7.82	9.02	15	3.35	8.61	10.03	17	3.33	11.93	14.51
2.00	7	2.91	2.81	2.58	7	2.92	2.99	2.84	8	3.01	3.33	3.24	9	2.92	4.82	5.25
*τ*	*n* = 10															
1.10	33	3.10	47.95	62.55	33	3.16	50.04	65.32	34	3.27	53.63	70.05	37	3.57	65.65	85.84
1.20	16	2.86	14.01	17.50	17	2.93	14.88	18.62	18	3.02	16.40	20.65	21	3.29	21.89	27.98
1.25	13	2.79	9.09	10.91	13	2.83	9.70	11.75	14	2.92	10.76	13.16	16	3.15	14.67	18.41
1.50	6	2.52	2.68	2.49	6	2.55	2.86	2.75	6	2.59	3.18	3.21	8	2.81	4.41	4.77
2.00	3	2.27	1.29	0.67	3	2.29	1.35	0.77	3	2.31	1.45	0.94	4	2.48	1.87	1.50

**Table 3:** Optimal charting parameters and the corresponding ARL1 and SDRL1 values of the multivariate SS chart for *p*∈{5,8}, *n*∈{6,10,15}, *τ*∈{0.10,1.20,1.25,1.50,2.00} and *γ*_0_∈{0.10,0.20,0.30,0.50}.

*τ*	*γ*_0_ = 0.10				*γ*_0_ = 0.20				*γ*_0_ = 0.30				*γ*_0_ = 0.50			
	L*	K*	*ARL*_1_	*SDRL*_1_	L*	K*	*ARL*_1_	*SDRL*_1_	L*	K*	*ARL*_1_	*SDRL*_1_	L*	K*	*ARL*_1_	*SDRL*_1_
*p* = 5															
*n* = 6															
1.10	71	4.46	112.56	147.69	71	4.50	115.14	151.09	72	4.57	119.65	157.02	74	4.68	135.50	177.77
1.20	47	4.23	49.41	63.96	48	4.27	51.28	66.45	49	4.33	54.58	70.88	52	4.42	66.83	87.27
1.25	39	4.12	35.90	45.98	40	4.16	37.44	48.03	42	4.23	40.20	51.68	46	4.33	50.65	65.71
1.50	21	3.76	12.39	14.78	22	3.80	13.13	15.73	23	3.85	14.49	17.54	27	3.94	19.97	24.86
2.00	11	3.37	4.73	4.93	12	3.43	5.09	5.35	13	3.48	5.77	6.21	16	3.56	8.64	9.97
*τ*	*n* = 10															
1.10	37	3.26	58.24	76.13	39	3.33	60.57	79.20	41	3.44	64.60	84.51	42	3.67	78.11	102.20
1.20	20	3.04	18.36	23.21	21	3.10	19.45	24.64	22	3.18	21.37	27.21	25	3.41	28.31	36.49
1.25	16	2.96	12.11	14.86	17	3.02	12.90	15.87	17	3.08	14.28	17.80	20	3.30	19.41	24.66
1.50	7	2.65	3.53	3.62	7	2.67	3.79	3.99	8	2.77	4.25	4.54	10	2.96	6.03	6.87
2.00	4	2.44	1.53	0.99	4	2.45	1.62	1.12	4	2.47	1.79	1.37	5	2.62	2.46	2.27
*τ*	*p* = 8															
	*n* = 10															
1.10	57	3.89	88.72	116.28	56	3.92	91.57	120.04	56	3.97	96.43	126.41	59	4.13	112.44	147.33
1.20	33	3.64	34.14	43.98	36	3.71	35.88	46.17	36	3.75	38.91	50.29	39	3.90	49.62	64.62
1.25	27	3.55	23.78	30.17	28	3.59	25.14	31.96	29	3.64	27.53	35.18	34	3.82	36.19	46.71
1.50	13	3.20	7.45	8.58	14	3.25	8.00	9.27	15	3.31	9.00	10.58	19	3.49	12.88	15.66
2.00	7	2.90	2.83	2.61	7	2.91	3.07	2.95	8	2.99	3.51	3.49	10	3.13	5.29	5.83
*τ*	*n* = 15															
1.10	31	3.08	48.01	62.65	34	3.14	50.24	65.57	37	3.23	54.07	70.61	38	3.41	66.71	87.25
1.20	17	2.88	14.03	17.45	17	2.91	14.97	18.75	18	2.97	16.61	20.94	20	3.14	22.48	28.84
1.25	13	2.78	9.11	10.93	14	2.84	9.76	11.75	14	2.88	10.92	13.37	16	3.05	15.12	19.04
1.50	6	2.52	2.69	2.50	6	2.53	2.88	2.78	6	2.56	3.24	3.29	8	2.75	4.59	5.03
2.00	3	2.27	1.29	0.67	3	2.28	1.35	0.78	3	2.30	1.47	0.98	4	2.46	1.96	1.63

From Tables 2 and 3, the proposed chart shows a better performance for larger values of *n*, with smaller values of L* and K*. For example, for (*p*,*n*,*τ*,*γ*_0_) = (2,5,1.10,0.10), (L*,K*,*ARL*_1_,*SDRL*_1_) = (47,3.60,74.72,97.85), but for (*p*,*n*,*τ*,*γ*_0_) = (2,10,1.10,0.10), (L*,K*,*ARL*_1_,*SDRL*_1_) = (31,3.04,44.09,57.46). Moreover, it is easier for larger values of *τ* to be detected, since larger values of *τ* show a larger shift from *γ*_0_. Thus, smaller *ARL*_1_ and *SDRL*_1_ values are associated with larger *τ*. Larger *τ* also results in smaller values of L*, and a smaller conforming region through smaller values of K*. For example, for (*p*,*n*,*τ*,*γ*_0_) = (2,5,1.10,0.10), (L*,K*,*ARL*_1_,*SDRL*_1_) = (47,3.60,74.72,97.85), but for (*p*,*n*,*τ*,*γ*_0_) = (2,5,2.00,0.10), (L*,K*,*ARL*_1_,*SDRL*_1_) = (5,2.64,2.10,1.75). Tables 2 and 3 also show that larger *γ*_0_ is associated with larger L*, K*, *ARL*_1_ and *SDRL*_1_, which shows a larger conforming region and weaker performance.

Table 4 illustrates the optimal (L*,K*) with its *EARL* for *p*∈{2,3,5,8} and *γ*_0_∈{0.10,0.20,0.30,0.50}, for scenarios in which the exact value of *τ* is unknown. Similar to Tables 2 and 3, for *p*∈{2,3}, *p* = 5 and *p* = 8, values of *n*∈{5,10}, *n*∈{6,10} and *n*∈{10,15}, respectively, are considered. To account for the uncertainty in the value of *τ*, we consider (*τ*_min_,*τ*_max_) = (1,2].

**Table 4:** Optimal charting parameters and the corresponding EARL values of the multivariate SS chart for *p*∈{2,3,5,8}, *n*∈{5,6,10,15} and *γ*_0_∈{0.10,0.20,0.30,0.50}.

*γ*_0_	L*	K*	*EARL*	L*	K*	*EARL*	L*	K*	*EARL*	L*	K*	*EARL*
	*p =* 2						*p =* 3					
	*n =* 5			*n =* 10			*n =* 5			*n =* 10		
0.10	26	3.36	19.63	21	2.91	11.75	30	3.60	23.68	22	2.97	12.68
0.20	27	3.44	20.24	21	2.97	12.24	30	3.66	24.41	22	3.02	13.20
0.30	26	3.53	21.33	21	3.05	13.08	30	3.73	25.71	22	3.10	14.11
0.50	25	3.48	25.55	20	3.26	15.96	29	3.70	30.88	21	3.29	17.25
*γ*_0_	*p =* 5						*p =* 8					
	*n =* 6			*n =* 10			*n =* 10			*n =* 15		
0.10	38	4.10	31.82	23	3.09	15.23	30	3.59	23.76	20	2.93	12.69
0.20	37	4.12	32.85	24	3.15	15.85	31	3.64	24.70	21	2.98	13.26
0.30	38	4.17	34.70	23	3.20	16.94	29	3.64	26.35	20	3.01	14.24
0.50	38	4.19	41.69	23	3.37	20.75	31	3.77	32.16	20	3.14	17.58

From Table 4, larger values of *p* result in larger L*, K* and *EARL*. This is consistent with the results in Tables 2 and 3. Similar to Tables 2 and 3, larger values of *n* result in smaller L*, K* and *EARL*, while larger *γ*_0_ results in larger *EARL*. In most cases, larger *γ*_0_ shows larger K*, however similar values of L* are observed for different values of *γ*_0_.

## 6. Comparisons

This section compares the proposed multivariate SS chart with the multivariate NSS, MEWMA and Shewhart *γ* charts. Similar numerical examples in Section 4 are adopted, but due to space constraint, we only consider *γ*_0_∈{0.10,0.50} in the comparison with the multivariate NSS chart, while *γ*_0_ = 0.10 is considered in the comparison with the MEWMA and Shewhart *γ* charts. Table 5 shows the *ARL*_1_ and *SDRL*_1_ comparisons with the multivariate NSS chart, while Table 6 compares with the MEWMA and Shewhart *γ* charts. Finally, Tables 7 and 8 show the *EARL* comparisons.

**Table 5:** A comparison of the ARL1 and SDRL1 of the multivariate SS and NSS charts for *p*∈{2,3,5,8}, *n*∈{5,6,10,15}, *τ*∈{1.10,1.20,1.25,1.50,2.00} and *γ*_0_∈{0.10,0.50}.

*τ*	*p* = 2								*p* = 3							
	*γ*_0_ = 0.10				*γ*_0_ = 0.50				*γ*_0_ = 0.10				*γ*_0_ = 0.50			
	SS		NSS		SS		NSS		SS		NSS		SS		NSS	
	*ARL*_1_	*SDRL*_1_	*ARL*_1_	*SDRL*_1_	*ARL*_1_	*SDRL*_1_	*ARL*_1_	*SDRL*_1_	*ARL*_1_	*SDRL*_1_	*ARL*_1_	*SDRL*_1_	*ARL*_1_	*SDRL*_1_	*ARL*_1_	*SDRL*_1_
	*n* = 5															
1.10	74.72	97.85	127.23	167.02	94.08	123.16	157.61	206.57	88.50	115.98	143.19	188.00	109.31	143.16	172.06	225.50
1.20	26.36	33.71	45.77	58.98	37.39	48.49	67.79	88.52	34.01	43.73	57.65	74.72	47.33	61.58	82.26	107.65
1.25	17.90	22.41	30.11	38.21	26.33	33.80	47.18	61.15	23.67	30.00	39.40	50.36	34.28	44.27	59.65	77.65
1.50	5.36	5.92	7.57	8.56	8.55	10.14	13.47	16.40	7.40	8.52	10.66	12.54	11.93	14.51	19.02	23.63
2.00	2.10	1.75	2.49	2.21	3.38	3.39	4.51	4.82	2.81	2.58	3.42	3.28	4.82	5.25	6.68	7.50
*τ*	*n* = 10															
1.10	44.09	57.46	82.94	108.44	60.80	79.48	117.18	153.76	47.95	62.55	89.09	116.59	65.65	85.84	124.11	162.88
1.20	12.50	15.49	21.66	27.23	19.59	24.96	36.87	47.51	14.01	17.50	24.42	30.88	21.89	27.98	41.21	53.31
1.25	8.07	9.57	13.10	15.97	13.01	16.20	23.27	29.45	9.09	10.91	14.91	18.33	14.67	18.41	26.40	33.62
1.50	2.41	2.19	3.08	2.97	3.88	4.11	5.59	6.25	2.68	2.49	3.49	3.55	4.41	4.77	6.47	7.40
2.00	1.22	0.55	1.31	0.70	1.69	1.23	2.01	1.61	1.29	0.67	1.40	0.86	1.87	1.50	2.27	1.99
*τ*	*p* = 5								*p* = 8							
	*γ*_0_ = 0.10				*γ*_0_ = 0.50				*γ*_0_ = 0.10				*γ*_0_ = 0.50			
	SS		NSS		SS		NSS		SS		NSS		SS		NSS	
	*ARL*_1_	*SDRL*_1_	*ARL*_1_	*SDRL*_1_	*ARL*_1_	*SDRL*_1_	*ARL*_1_	*SDRL*_1_	*ARL*_1_	*SDRL*_1_	*ARL*_1_	*SDRL*_1_	*ARL*_1_	*SDRL*_1_	*ARL*_1_	*SDRL*_1_
	*n* = 6								*n* = 10							
1.10	112.56	147.69	167.13	219.54	135.50	177.77	195.18	256.02	88.72	116.28	141.81	186.24	112.44	147.33	175.16	229.82
1.20	49.41	63.96	79.65	103.79	66.83	87.27	107.38	140.77	34.14	43.98	57.73	74.83	49.62	64.62	85.19	111.45
1.25	35.90	45.98	57.32	74.02	50.65	65.71	82.28	107.43	23.78	30.17	39.47	50.46	36.19	46.71	62.19	80.89
1.50	12.39	14.78	18.05	21.90	19.97	24.86	31.30	39.53	7.45	8.58	10.73	12.64	12.88	15.66	20.39	25.28
2.00	4.73	4.93	5.97	6.40	8.64	9.97	12.24	14.47	2.83	2.61	3.45	3.32	5.29	5.83	7.33	8.32
*τ*	*n* = 15								*n* = 15							
1.10	58.24	76.13	104.27	136.71	78.11	102.20	140.18	183.96	48.01	62.65	89.02	116.48	66.71	87.25	125.32	164.50
1.20	18.36	23.21	32.19	41.08	28.31	36.49	52.72	68.46	14.03	17.45	24.39	30.85	22.48	28.84	42.21	54.56
1.25	12.11	14.86	20.21	25.21	19.41	24.66	35.08	45.07	9.11	10.93	14.90	18.31	15.12	19.04	27.21	34.66
1.50	3.53	3.62	4.78	5.10	6.03	6.87	9.19	10.86	2.69	2.50	3.49	3.47	4.59	5.03	6.77	7.72
2.00	1.53	0.99	1.72	1.27	2.46	2.27	3.14	3.05	1.29	0.67	1.40	0.86	1.96	1.63	2.40	2.18

**Table 6:** A comparison of the ARL1 and SDRL1 of the multivariate SS chart with the MEWMA and Shewhart *γ* charts for *γ*_0_ = 0.10, *p*∈{2,3,5,8}, *n*∈{5,6,10,15} and *τ*∈{1.10,1.20,1.25,1.50,2.00}.

*τ*	*p* = 2						*p* = 3					
	SS		MEWMA		Shewhart		SS		MEWMA		Shewhart	
	*ARL*_1_	*SDRL*_1_	*ARL*_1_	*SDRL*_1_	*ARL*_1_	*SDRL*_1_	*ARL*_1_	*SDRL*_1_	*ARL*_1_	*SDRL*_1_	*ARL*_1_	*SDRL*_1_
	*n* = 5											
1.10	74.72	97.85	56.54	47.47	171.28	170.78	88.50	115.98	70.37	61.35	187.38	186.88
1.20	26.36	33.71	220	16.94	75.36	74.86	34.01	43.73	29.57	23.03	90.65	90.15
1.25	17.90	22.41	16.90	12.09	52.50	52.00	23.67	30.00	22.08	16.56	65.53	65.03
1.50	5.36	5.92	6.74	4.62	13.79	13.29	7.40	8.52	8.96	6.35	19.02	18.52
2.00	2.10	1.75	2.86	1.92	3.79	3.25	2.81	2.58	3.80	2.70	5.36	4.84
*τ*	*n* = 10											
1.10	44.09	57.46	31.68	23.75	122.37	121.87	47.95	62.55	34.48	26.30	129.25	128.75
1.20	12.50	15.49	11.86	7.94	40.66	40.16	14.01	17.50	13.01	8.84	44.94	44.44
1.25	8.07	9.57	8.66	5.61	25.68	25.18	9.09	10.91	9.51	6.25	28.78	28.28
1.50	2.41	2.19	3.37	2.09	5.51	4.99	2.68	2.49	3.70	2.34	6.32	5.80
2.00	1.22	0.55	1.52	0.81	1.69	1.08	1.29	0.67	1.65	0.92	1.87	1.27
*τ*	*p* = 5						*p* = 8					
	SS		MEWMA		Shewhart		SS		MEWMA		Shewhart	
	*ARL*_1_	*SDRL*_1_	*ARL*_1_	*SDRL*_1_	*ARL*_1_	*SDRL*_1_	*ARL*_1_	*SDRL*_1_	*ARL*_1_	*SDRL*_1_	*ARL*_1_	*SDRL*_1_
	*n* = 6						*n* = 10					
1.10	112.56	147.69	94.06	83.90	209.40	208.90	88.72	116.28	70.52	61.14	184.80	184.30
1.20	49.41	63.96	43.80	35.31	116.90	116.40	34.14	43.98	29.69	23.13	90.59	90.09
1.25	35.90	45.98	33.57	26.25	88.35	87.85	23.78	30.17	22.17	16.65	65.52	65.02
1.50	12.39	14.78	14.38	10.53	30.53	30.03	7.45	8.58	9.01	6.38	19.14	18.63
2.00	4.73	4.93	6.48	4.53	9.42	8.91	2.83	2.61	3.82	2.72	5.41	4.89
*τ*	*n* = 10						*n* = 15					
1.10	58.24	76.13	42.40	33.70	145.84	145.34	48.01	62.65	34.58	26.44	128.92	128.42
1.20	18.36	23.21	16.37	11.52	56.46	55.95	14.03	17.45	13.04	8.87	44.78	44.28
1.25	12.11	14.86	12.02	8.16	37.44	36.94	9.11	10.93	9.53	6.27	28.69	28.19
1.50	3.53	3.62	4.71	3.08	8.79	8.27	2.69	2.50	3.71	2.34	6.33	5.81
2.00	1.53	0.99	2.04	1.24	2.46	1.89	1.29	0.67	1.65	0.92	1.28	1.28

**Table 7:** A comparison of the EARL of the multivariate SS and NSS charts for *p*∈{2,3,5,8}, *n*∈{5,6,10,15} and *γ*_0_∈{0.10,0.50}, where (*τ*_min_,*τ*_max_) = (1,2].

*γ*_0_	*p* = 2				*p* = 3			
	*n* = 5		*n* = 10		*n* = 5		*n* = 10	
	SS	NSS	SS	NSS	SS	NSS	SS	NSS
0.10	19.63	30.64	11.75	19.61	23.68	35.86	12.68	20.95
0.50	25.55	40.76	15.96	27.46	30.88	47.65	17.25	29.35
*γ*_0_	*p* = 5				*p* = 8			
	*n* = 6		*n* = 10		*n* = 10		*n* = 15	
	SS	NSS	SS	NSS	SS	NSS	SS	NSS
0.10	31.82	45.93	15.23	24.53	23.76	35.75	12.69	20.99
0.50	41.69	61.06	20.75	34.30	32.16	49.25	17.58	29.80

**Table 8:** A comparison of the EARL of the multivariate SS chart with the MEWMA and Shewhart *γ* charts for *γ*_0_ = 0.10, *p*∈{2,3,5,8} and *n*∈{5,6,10,15}, where (*τ*_min_,*τ*_max_) = (1,2].

*n*	SS	MEWMA	Shewhart	*n*	SS	MEWMA	Shewhart
	*p* = 2				*p* = 3		
5	19.63	17.20	42.54	5	23.68	21.28	49.47
10	11.75	10.13	27.48	10	12.68	10.92	29.30
*n*	*p* = 5			*n*	*p* = 8		
6	31.82	29.59	62.36	10	23.76	21.36	49.19
10	15.23	13.16	34.14	15	12.69	10.95	29.34

Table 5 shows that the multivariate SS chart has a smaller *ARL*_1_ and *SDRL*_1_ values than the multivariate NSS chart, particularly when *τ* is small. This results in quicker detection of the assignable cause(s) and less variability in the run lengths. For example, for (*p*,*n*,*τ*,*γ*_0_) = (2,5,1.10,0.10), the (*ARL*_1_,*SDRL*_1_) = (74.72,97.85) for the multivariate SS chart, while the (*ARL*_1_,*SDRL*_1_) = (127.23,167.02) for the multivariate NSS chart. A smaller improvement is shown for larger *τ*. For example, for (*p*,*n*,*τ*,*γ*_0_) = (2,5,2.00,0.10), the (*ARL*_1_,*SDRL*_1_) = (2.10,1.75) for the multivariate SS chart, while (*ARL*_1_,*SDRL*_1_) = (2.49,2.21) for the multivariate NSS chart. Thus, the improvement is not as large as that for *τ* = 1.10. The multivariate SS chart also shows a larger improvement for smaller values of *n*, and larger values of *p* and *γ*_0_.

Tables 7 and 8 show the *EARL* comparisons, where (*τ*_min_,*τ*_max_) = (1,2]. Table 7 shows the multivariate SS chart has a smaller *EARL* than the multivariate NSS chart. For example, for (*p*,*n*,*γ*_0_) = (2,5,0.10), the *EARL* = 19.63 for the multivariate SS chart, while *EARL* = 30.64 for the multivariate NSS chart. Similar to Table 5, smaller *n*, and larger *p* and *γ*_0_ shows larger improvement. Compared to the MEWMA and Shewhart *γ* charts, as shown in Table 8, the multivariate SS chart significantly outperforms the Shewhart *γ* chart, whereas the MEWMA *γ* chart slightly outperforms the multivariate SS chart.

## 7. An illustrative example

The implementation of the multivariate SS chart on an illustrative example that was also adopted by Giner-Bosch et al. [22] is shown in this section. In this example, the *γ* for the investment returns from *p* = 3 industrial sectors *S*_1_ (automotive), *S*_2_ (aeronautic) and *S*_3_ (electronic) for *n* = 5 regions *R*_1_ (Africa), *R*_2_ (North America), *R*_3_ (South America), *R*_4_ (Asia) and *R*_5_ (Europe) are monitored. Table 9 shows the rates of return from years 2000 to 2016, and for each of these years, the $\bar{X}$, **S** and ${\hat{\gamma }}^{2}$ are shown.

**Table 9:** Rates of return (in %) from 2000 to 2016 for *p* = 3 industrial sectors {*S*_1_,*S*_2_,*S*_3_} and *n* = 5 regions {*R*_1_,*R*_2_,*R*_3_,*R*_4_,*R*_5_}.

		*R*_1_	*R*_2_	*R*_3_	*R*_4_	*R*_5_	$\bar{X}$	S			${\hat{\gamma }}^{2}$
2000	*S*_1_	17.8	25.2	18.1	19.0	19.0	19.82	9.3320	1.3540	0.1205	0.004082
	*S*_2_	42.0	40.7	35.5	42.0	40.5	40.14	1.3540	7.2230	1.1710	
	*S*_3_	8.3	9.4	8.6	10.5	12.1	9.78	0.1205	1.1710	2.4070	
2001	*S*_1_	21.5	22.5	22.0	18.1	19.1	20.64	3.7180	3.6455	0.0435	0.001739
	*S*_2_	40.5	36.9	42.0	36.2	35.1	38.14	3.6455	8.7530	4.1410	
	*S*_3_	11.9	8.5	12.8	11.4	9.3	10.78	0.0435	4.1410	3.2770	
2002	*S*_1_	17.5	18.9	19.1	21.8	22.7	20.00	4.7000	-0.5675	0.3900	0.000539
	*S*_2_	39.9	38.2	38.0	39.8	37.8	38.74	-0.5675	1.0480	-0.0355	
	*S*_3_	8.9	11.0	7.8	9.6	9.5	9.36	0.3900	-0.0355	1.3530	
2003	*S*_1_	19.1	18.7	21.4	20.8	18.9	19.78	1.5170	0.5765	0.4550	0.001422
	*S*_2_	38.8	42.4	42.5	39.6	39.5	40.56	0.5765	3.0730	0.6325	
	*S*_3_	9.7	10.3	11.0	10.4	10.1	10.3	0.4550	0.6325	0.2250	
2004	*S*_1_	19.0	21.6	19.5	19.0	19.2	19.66	1.2180	1.2920	-0.1385	0.002000
	*S*_2_	39.4	40.8	35.9	35.0	41.6	38.54	1.2920	8.6780	-2.0265	
	*S*_3_	8.2	9.7	12.6	10.5	10.9	10.38	-0.1385	-2.0265	2.6070	
2005	*S*_1_	18.9	19.1	21.3	17.0	20.2	19.30	2.5750	3.3900	-0.6550	0.001470
	*S*_2_	41.1	38.1	42.8	36.2	39.2	39.48	3.3900	6.5970	-0.1985	
	*S*_3_	10.8	9.5	8.7	9.6	9.2	9.56	-0.6550	-0.1985	0.6030	
2006	*S*_1_	17.9	20.0	20.5	18.5	19.4	19.26	1.1330	-1.6770	-0.5400	0.000603
	*S*_2_	43.0	41.2	36.5	39.1	41.0	40.16	-1.6770	6.0930	2.0675	
	*S*_3_	8.2	9.6	6.3	9.6	9.8	8.70	-0.5400	2.0675	2.2100	
2007	*S*_1_	20.6	18.7	18.5	23.6	19.7	20.22	4.2770	3.6525	3.8680	0.001834
	*S*_2_	40.3	36.9	35.4	40.8	41.6	39.00	3.6525	7.2650	3.9925	
	*S*_3_	9.0	8.7	6.8	12.4	9.5	9.28	3.8680	3.9925	4.0870	
2008	*S*_1_	19.0	20.4	21.6	20.3	18.4	19.94	1.5880	1.0640	1.3085	0.001383
	*S*_2_	37.3	44.8	40.5	38.9	40.6	40.42	1.0640	7.8170	4.2605	
	*S*_3_	8.3	11.7	10.7	7.0	8.2	9.18	1.3085	4.2605	3.7870	
2009	*S*_1_	21.2	16.5	18.2	21.2	21.2	19.66	4.8080	1.9075	0.2025	0.001305
	*S*_2_	38.9	39.6	36.8	40.6	41.6	39.50	1.9075	3.3200	-0.9650	
	*S*_3_	10.9	8.6	9.1	7.0	8.9	8.90	0.2025	-0.9650	1.9350	
2010	*S*_1_	9.6	8.8	8.4	6.9	7.4	8.22	1.1720	-2.0755	-2.4445	0.000499
	*S*_2_	19.5	17.9	18.9	23.7	21.6	20.32	-2.0755	5.4020	4.0830	
	*S*_3_	2.2	5.0	5.3	8.9	6.0	5.48	-2.4445	4.0830	5.7470	
2011	*S*_1_	11.0	11.8	15.6	11.2	10.3	11.98	4.3820	-1.7770	-0.5615	0.002599
	*S*_2_	18.9	21.6	19.0	20.1	22.5	20.42	-1.7770	2.5370	0.7490	
	*S*_3_	4.6	6.2	4.8	6.1	5.5	5.44	-0.5615	0.7490	0.5330	
2012	*S*_1_	9.5	10.0	8.3	8.8	12.3	9.78	2.4070	2.2845	-0.6710	**0.007852**
	*S*_2_	22.1	17.1	19.6	23.4	25.2	21.48	2.2845	10.1570	1.0565	
	*S*_3_	4.5	3.8	6.2	5.8	5.0	5.06	-0.6710	1.0565	0.9380	
2013	*S*_1_	10.0	8.0	8.2	11.5	7.1	8.96	3.1230	-1.5155	-1.8780	0.001588
	*S*_2_	21.1	21.0	21.2	17.9	20.0	20.24	-1.5155	1.9430	2.0330	
	*S*_3_	4.4	7.0	7.3	3.0	4.0	5.14	-1.8780	2.0330	3.6380	
2014	*S*_1_	12.2	9.6	7.7	11.3	13.2	10.80	4.7550	-1.3900	-0.4325	**0.004144**
	*S*_2_	20.0	18.2	18.4	19.6	14.8	18.20	-1.3900	4.2000	0.2850	
	*S*_3_	4.6	4.5	4.4	3.5	3.8	4.16	-0.4325	0.2850	0.2330	
2015	*S*_1_	11.6	9.8	12.4	11.0	9.4	10.84	1.5480	0.6550	0.7060	0.003456
	*S*_2_	17.2	20.1	21.5	18.6	18.1	19.10	0.6550	2.9050	1.8425	
	*S*_3_	4.6	6.4	6.4	4.7	3.3	5.08	0.7060	1.8425	1.7570	
2016	*S*_1_	11.2	5.4	9.4	8.4	6.9	8.26	4.9980	-0.5210	1.9820	**0.006183**
	*S*_2_	20.0	22.1	24.5	17.2	20.6	20.88	-0.5210	7.2470	1.8835	
	*S*_3_	5.8	4.0	7.3	4.7	4.9	5.34	1.9820	1.8835	1.6130	

The coefficient of variation measures the volatility (standard deviation) of investment returns compared to its expected return. Hence, monitoring *γ* allows investors to monitor the relative risk of investments, in order to make a fair comparison between different investments. Suppose the company feels that the rates of return and relative risk for years 2000 to 2009 are satisfactory. Thus, the rates of return from years 2000 to 2009 are considered as the Phase I samples, and $\gamma_{0}^{2}$ are estimated from the average of the ${\hat{\gamma }}^{2}$ from years 2000 to 2009, i.e.,

$${\hat{\gamma }}_{0}^{2}=\frac{0.004082+0.001739+\ldots +0.001305}{10}=0.00163769.$$ (25)

The company would like to monitor whether there is any shift in the relative risks of the investments from years 2010 to 2016. Suppose the company is not sure what is the size of shift that needs to be detected. In this case, the optimal (L*,K*) for the multivariate SS chart will be determined from the second approach as described in Section 3, i.e., the (L*,K*) in minimizing the *EARL*, subject to constraints in the *ARL*_0_, will be adopted to monitor the relative risks of the investment returns from years 2010 to 2016.

**Fig 3:**
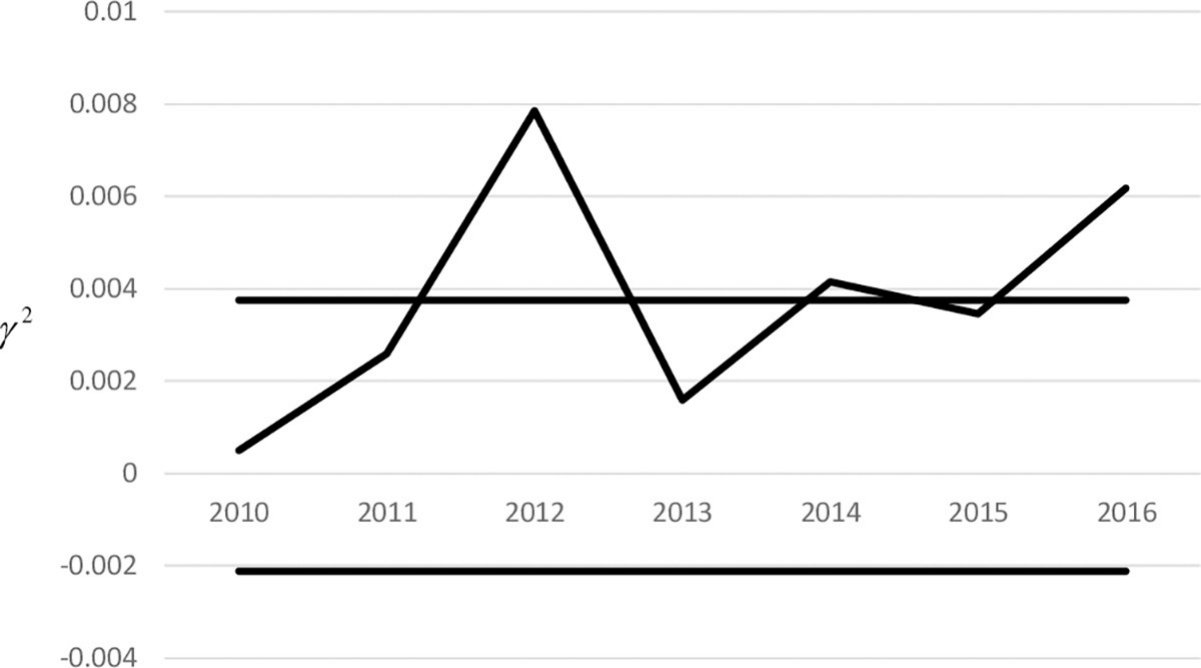
*γ*^2^ Sub–chart of the SS chart for monitoring the investment returns from years 2010 to 2016.

By adopting the second approach for $\left(p,n,\tau_{\min},\tau_{\max},{\hat{\gamma }}_{0})=(3,5,1,2,0.0404684\right)$, (L*,K*) = (30,3.59) is obtained, with an *EARL* of 23.49. Fig 3 shows the *γ*^2^ sub-chart of the multivariate SS chart that monitors the ${\hat{\gamma }}^{2}$ of the investment returns from years 2010 to 2016.

From Fig 3, the ${\hat{\gamma }}^{2}$ for years 2012, 2014 and 2016 is larger than the *UCL*. Hence, they are non-conforming samples. The CRL for each of these samples are: CRL_1_ = 3, CRL_2_ = 2 and CRL_3_ = 2, all of which are less than L*. Thus, the samples for years 2012, 2014 and 2016 are out-of-control samples. The multivariate SS chart shows an increase in the relative risk for the companies’ investments in years 2012, 2014 and 2016. This agrees with the results from Giner-Bosch et al. [22] who showed that there is a change in the rates of returns from years 2012 onwards. Note that Giner-Bosch et al. [22] monitored the investment returns through the MEWMA chart.

## 8. Conclusion

A multivariate SS chart to monitor *γ* is proposed in this paper. Formulae for the *ARL*, *SDRL* and *EARL* criteria are derived, and algorithms are proposed for the optimization of the proposed multivariate SS chart. Tables of optimal charting parameters and performance are shown for numerical examples with different *p*, *n*, *τ* and *γ*_0_ values, and also for unknown *τ*. The multivariate SS chart is shown to outperform the multivariate NSS chart. A larger improvement is shown for smaller *τ* and *n*, and larger *p* and *γ*_0_. The multivariate SS chart significantly outperforms the Shewhart *γ* chart, and shows marginally better performance than the MEWMA chart for moderate and large *τ*. The proposed multivariate SS chart provides a good alternative for practitioners.

The proposed multivariate SS chart adopts fixed charting parameters. In the future, a multivariate SS chart with adaptive charting parameters can be developed. Another possible area of research is to evaluate the multivariate SS chart through its *MRL* and run length percentiles, to account for skewed run length distributions.

## References

[1] Wu Z, Spedding TA. A synthetic control chart for detecting small shifts in the process mean. *J Qual Tech*. 2000; 32: 32–38.

[2] Rakitzis AC, Chakraborti S, Shongwe SC, Graham MA, Khoo MBC. An overview of synthetic‐type control charts: Techniques and methodology. *Qual Reliab Eng Int*. 2019; 35: 2081–2096.

[3] Lee MH, Khoo MBC, Chew XY, Then PHH. Economic-statistical design of synthetic np chart with estimated process parameter. *Plos One*. 2020; 15: e0230994. doi: 10.1371/journal.pone.0230994 32267874 PMC7141697

[4] Haq A, Khoo MBC. A synthetic double sampling control chart for process mean using auxiliary information. *Qual Reliab Eng Int*. 2019; 35: 1803–1825.

[5] Hu XL, Tang AA, Qiao YL, Sun JS, Guo BC. On the conditional performance of the synthetic chart with unknown process parameters using the exceedance probability criterion. *Plos One*. 2020; 15: e0239538. doi: 10.1371/journal.pone.0239538 33017409 PMC7535069

[6] Haq A. A new nonparametric synthetic EWMA control chart for monitoring process mean. *Commun Stat Simulat*. 2019; 16: 1665–1676.

[7] Calzada ME, Scariano SM. A synthetic control chart for the coefficient of variation. *J Stat Comput Sim*. 2013; 83: 853–867.

[8] Kang CW, Lee MS, Seong YJ, Hawkins DM. A control chart for the coefficient of variation. *J Qual Tech*. 2007; 39: 151–158.

[9] Castagliola P, Celano G, Psarakis S. Monitoring the coefficient of variation using EWMA charts. *J Qual Tech*. 2011; 43: 249–265.

[10] Tran KP, Nguyen HD, Nguyen QT, Chattinnawat W. One-sided synthetic control charts for monitoring the coefficient of variation with measurement errors. *IEEE In C Ind Eng Eng Man*. 2018; 1667–1671.

[11] Yeong WC, Lim SL, Khoo MBC, Chew MH, Alex LJX. The economic and economic-statistical designs of the synthetic chart for the coefficient of variation. *J Test Eval*. 2018; 46: 1175–1195.

[12] Yeong WC, Lee PY, Lim SL, Khaw KW, Khoo MBC. A side-sensitive synthetic coefficient of variation chart. *Qual Reliab Eng Int*. 2021; 37: 2014–2033.

[13] Yeong WC, Lee PY, Lim SL, Ng PS, Khaw KW. Optimal designs of the side sensitive synthetic chart for the coefficient of variation based on the median run length and expected median run length. *Plos One*. 2021; 16: e0255366. doi: 10.1371/journal.pone.0255366 34329357 PMC8323885

[14] Yeong WC, Khoo MBC, Teoh WL, Castagliola P. A control chart for the multivariate coefficient of variation. *Qual Reliab Eng Int*. 2016; 32: 1213–1225.

[15] Lim AJX, Khoo MBC, Teoh WL, Haq A. Run sum chart for monitoring multivariate coefficient of variation. *Comput Ind Eng*. 2017; 109: 84–95.

[16] Abbasi SA, Adegoke NA. Multivariate coefficient of variation control charts in phase I of SPC. *Int J Adv Manuf Tech*. 2018; 99: 1903–1916.

[17] Khaw KW, Khoo MBC, Castagliola P, Rahim MA. New adaptive control charts for monitoring the multivariate coefficient of variation. *Comput Ind Eng*. 2018; 126: 595–610.

[18] Chew XY, Khoo MBC, Khaw KW, Yeong WC, Chong ZL. A proposed variable parameter control chart for monitoring the multivariate coefficient of variation. *Qual Reliab Eng Int*. 2019; 35: 2442–2461.

[19] Nguyen QT, Tran KP, Heuchenne HL, Nguyen TH, Nguyen HD. Variable sampling interval Shewhart control charts for monitoring the multivariate coefficient of variation. *Appl Stoch Model Bus*. 2019; 35: 1253–1268.

[20] Ayyoub HN, Khoo MBC, Sajal S, Lee MH. Variable sampling interval EWMA chart for multivariate coefficient of variation. *Commun Stat Theory*. 2020; in-press.

[21] Khatun M, Khoo MBC, Lee MH, Castagliola P. One-sided control charts for monitoring the multivariate coefficient of variation in short production runs. *T I Meas Control*. 2018; 41: 1712–1728.

[22] Giner-Bosch V, Tran KP, Castagliola P, Khoo MBC. An EWMA control chart for the multivariate coefficient of variation. *Qual Reliab Eng Int*. 2019; 35: 1515–1541.

[23] Haq A, Khoo MBC. New adaptive EWMA control charts for monitoring univariate and multivariate coefficient of variation. *Comput Ind Eng*. 2019; 131: 28–40.

[24] Chew XY, Khaw KW, Yeong WC. The efficiency of run rules schemes for the multivariate coefficient of variation: a Markov chain approach. *J Appl Stat*. 2020; 47: 460–480. doi: 10.1080/02664763.2019.1643296 35706968 PMC9042138

[25] Chew XY, Khaw KW, Lee MH. The efficiency of run rules schemes for the multivariate coefficient of variation in short runs process. *Commun Stat Simulat*. 2019; in-press.

[26] Ayyoub HN, Khoo MBC, Sajal S, Castagliola P. Multivariate coefficient of variation charts with measurement errors. *Comput Ind Eng*. 2020; 147: 106633.

[27] Ayyoub HN, Khoo MBC, Lee MH, Haq A. Monitoring multivariate coefficient of variation with upward Shewhart and EWMA charts in the presence of measurement errors using the linear covariate error model. *Qual Reliab Eng Int*. 2021; 37: 694–716.

[28] Nguyen QT, Giner-Bosch V, Tran KD, Heuchenne C, Tran KP. One-sided variable sampling interval EWMA control charts for monitoring the multivariate coefficient of variation in the presence of measurement errors. *Int J Adv Manuf Tech*. 2021; 115: 1821–1851.

[29] Voinov VG, Nikulin MS. *Unbiased Estimators and Their Applications*, *Multivariate Case*, vol. 2. Kluwer: Dordrecht, 1996.

[30] Piessens R, de Doncker-Kapenga E, Uberhuber CW, Kahaner D. *Quadpack*. *A subroutine package for automatic integration*, Springer Series in Computational Mathematics. Springer-Verlag: Berlin, Heildelberg, 1983.

[31] Kovvali N. Theory and applications of Gaussian quadrature methods. *Synthesis Lectures on Algorithms and Software in Engineering*. 2011; 3: 1–65.

